# Associations of measured resting energy expenditure with predictive equations, NUTRIC score, and patient outcomes

**DOI:** 10.1186/s43162-021-00060-1

**Published:** 2021-10-18

**Authors:** Elham Sobhy, Radwa Abdel Kader, Alshaimaa Aboulfotouh, Mohammed Eshra, Mohamed Sayed

**Affiliations:** 1 Internal Medicine Department, KasrAlainy Cairo University, Cairo, Egypt; 2 Neurology Department, KasrAlainy Cairo University, Cairo, Egypt; 3 Physiology Department, KasrAlainy Cairo University, Cairo, Egypt; 4 Giza, Egypt

**Keywords:** REE, Indirect calorimetry, NUTRIC score, Weight-based formula, PSU_m_, Faisy–Fagon

## Abstract

**Background:**

Indirect calorimetry is the reference method for measuring resting energy expenditure (REE), but the necessary equipment and technical expertise are not always available. Meanwhile, the NUTrition Risk in the Critically ill (NUTRIC) scale is designed to identify patients who would benefit from nutrition therapy, but no data are available regarding the association of NUTRIC scores with REE. Several predictive formulas are available as alternatives to indirect calorimetry for calculation of energy requirements, but they have not been compared in a homogeneous group of critically ill patients. The purpose of the study is to examine the correlations between energy expenditure and NUTRIC scores or patient outcomes, and to compare measured REE with estimations of energy expenditure.

**Methods:**

In this observational, prospective study, indirect calorimetry was performed on 50 mechanically ventilated patients. Energy expenditure was also estimated with the bodyweight-based, Faisy–Fagon, and Penn-State PSU_m_ equations.

**Results:**

REE was higher in patients who survived treatment than in those who died, and was positively correlated with length of stay and duration of ventilation. NUTRIC scores did not correlate with REE. The Faisy–Fagon equation overestimated expenditure, whereas PSU_m_ was unbiased and accurate. Calculations based on 25 kcal/kg bodyweight/day overestimated expenditure, whereas 23 kcal/kg/day produced unbiased estimates with greater accuracy than PSU_m_.

**Conclusion:**

REE was positively associated with patient outcomes. Energy expenditure was accurately predicted by calculations of 23 kcal/kg bodyweight/day.

## Background

Critically ill patients are considered to be at a high nutritional risk in terms of their assessment scores or risk-assessment outcomes [1]. Meeting caloric demand with adequate nutrition in such patients is related to positive outcomes. However, the optimal number of calories that should be prescribed to a critically ill patient is a matter of debate [2].

The NUTrition Risk in the Critically ill (NUTRIC) scoring system is designed to quantify the risk of developing adverse events in critically ill patients, in whom risks might be modified by aggressive nutrition therapy. NUTRIC has been validated for the intensive-care unit (ICU) outcomes weaning from mechanical ventilation, length of stay (LOS) and mortality [3, 4]. However, there are no sufficient information on the relationship between NUTRIC scores and resting energy expenditure (REE) of critically ill patients, or on the relationship between REE and patient outcomes.

Although indirect calorimetry is considered the gold standard for measurement of REE, the necessary equipment and technical expertise are not always available. Additionally, REE measurements should ideally be performed under standardized conditions that are not always feasible for patients in ICUs. As a consequence, predictive formulas that enable estimation of REE have been developed for use in ICUs [5].

The American College of Chest Physicians (ACCP) published a consensus statement in 1997 on nutrition in the critical-care setting, stating that an energy expenditure of 25 kcal/kg bodyweight/day is a reasonable standard for most patients [6]. However, subsequent testing of the equation in mixed surgical and medical patients has shown this figure to be biased, inaccurate, and imprecise [7].

The Penn State equation (PSU_m_) was developed using a mixed ICU population of 169 participants in Pennsylvania [8, 9]. The equation was found to be unbiased, accurate, and precise, although it was considered possible that the performance of the equation might vary according to the level of severity of the illness [7].

The Faisy–Fagon equation was presented in 2003 following its development from the analysis of data relating to 70 mechanically ventilated adult critical-care patients in France [10]. The equation was subsequently validated in a further 45 mechanically ventilated patients and found to be accurate and unbiased [11].

The 202 ventilated critical-care patients included in one of the largest studies to validate multiple energy expenditure equations [7] were a mix of medical and surgical patients with no recorded Acute Physiologic Assessment and Chronic Health Evaluation (APACHE) scores, and the method used for measurement of bodyweight was not clear.

Because of the existence of multiple predictive equations, it is necessary to compare the performance of these equations in specific groups, such as in medical patients in whom active metabolic weights are calculated. In this study, our aims were to correlate REE measured by indirect calorimetry with NUTRIC scores and patient outcomes, and to compare REE with calorie requirements estimated with the bodyweight-based, Faisy–Fagon, and PSU_m_ equations.

## Methods

This prospective, observational study was conducted in the Internal Medicine ICU over a period of 8 months from August 2016 to April 2017. All patients ≥ 18 years old who were intubated and mechanically ventilated for 24 h and that were hemodynamically stable were considered for inclusion.

### Exclusion criteria

Potential participants were excluded on the basis of any of the following factors: hemodynamic instability (introduction, dose modification, or withdrawal of inotropic drugs during the preceding 2 h); respiratory instability (pulse oxygen saturation < 90%, modification of ventilator settings during the preceding 2 h and signs of hyperventilation (respiratory rate > 35/min); bicarbonate infusion, digestive or renal losses of bicarbonate (diarrhea, ureterosigmoidostomy, use of acetazolamide) or the use of extracorporeal blood lines for dialysis; intravenous carbohydrate load ≥ 15 kcal/kg/day; and air leaks in the respiratory system or fraction of inspired oxygen (FiO_2_) ≥ 60%.

### Methodology

Eligible patients were thoroughly examined. Mid-upper-arm circumference (MUAC) was measured to estimate body mass index (BMI) category. MUAC measurements were performed using the technique recommended by the British Association for Parenteral and Enteral Nutrition (BAPEN; Malnutrition Universal Screening Tool) in 2011. A MUAC < 23.5 cm was considered to indicate a BMI < 20 kg/m^2^, whereas a MUAC > 32.0 cm suggested BMI > 30 kg/m^2^. Height was estimated from the length of the forearm (ulna), using tables provided by the Malnutrition Advisory Group of BAPEN [12].

Weight was calculated from estimated BMI and height. Notably, it has since been determined that weight is strongly correlated with MUAC in adults [13], with a very nearly linear relationship that is defined by the equation weight (kg) = (MUAC [cm] × 4) − 50. This finding is supported by the median MUAC (30 cm) and weight (70 kg) values in our population.

Routine laboratory tests (complete blood count [CBC], liver function, kidney function, electrolytes, prothrombin time and lipid profile) were performed for all patients. The APACHE II score, Sequential Organ Failure Assessment (SOFA) score, and NUTRIC score were calculated for each patient.

### Estimation of caloric requirements

Three equations were used for estimation of energy requirements (kcal/day) of each participant. For the weight-based formula, caloric requirement = bodyweight (kg) × 25 [6]^.^ For the Faisy–Fagon equation, caloric requirement = (8 × bodyweight [kg]) + (14 × height [cm]) + (32 × minute ventilation [l/min]) + (94 × body temperature [°C]) − 4834 [10]^.^ For the PSU_m_ equation, caloric requirement = (0.96 × [value from Mifflin St Jeor equation]) + (167 × [maximum body temperature in the previous 24 h (°C)]) + (31 × minute ventilation [l/min]) – 6212. The Mifflin St Jeor equations were for men, (10 × weight [kg]) + (6.25 × height [cm]) − (5 × age [years]) + 5; and for women, (10 × weight [kg]) + (6.25 × height [cm]) − (5 × age [years]) − 161. For all equations, the weight used was the actual weight if the patient had a normal BMI (20–24.9), was overweight (BMI 25–29.9) or was underweight (BMI < 18.5), or was the adjusted weight ([ideal body weight] + 0.25 × ([actual weight] − [ideal weight])) in obese patients (BMI > 30).

Indirect calorimetry was carried out using a metabolic module connected to Carescape R860 ventilator, with measurement of respiratory gas exchange for 30 min, and calculation of REE by the modified Weir formula: (5.5 × VO_2_ [ml/min]) + (1.76 × VCO_2_ [ml/min]) − (1.99 × UN (g/day), where UN is urea nitrogen excretion and is assumed to be 13 g/day for adults.

Our study was approved by the ethical committee.

### Statistical methods

All data were entered into a Microsoft Excel spreadsheet. Data were coded and entered using the Statistical Package for the Social Sciences (SPSS) version 25. Comparisons between quantitative variables were performed using the unpaired *t* test or the nonparametric Mann–Whitney test. Paired measurements for each patient were used in a paired *t* test [14]. Correlations between quantitative variables were determined with the Spearman correlation coefficient [15]. *P* values < 0.05 were considered statistically significant.

## Results

Fifty mechanically ventilated patients were included in the study, 56% of whom were female. Further demographic and descriptive data for the participants are shown in Table 1, and laboratory data are shown in Table 2.

**Table 1:** Demographic and descriptive data of the study participants

Characteristic	Mean ± SD	Median (range)
Age (years)	56.44 ± 19.21	62.50 (22–86)
Height (cm)	169.36 ± 5.51	170.0 (155–182)
Mid-upper arm circumference (cm)	29.34 ± 4.61	30.00 (21–41)
Bodyweight (kg)	70.22 ± 9.71	70.00 (53–114)
BMI (kg/m^2)^	27.29 ± 5.08	27.50 (16–39)
Temperature (°C)	37.32 ± 0.59	37.00 (37–39)
MV (L/min)	9.26 ± 2.57	8.00 (6–15)
APACHE II score	16.60 ± 7.45	16.00 (3–35)
SOFA score	5.00 ± 3.08	5.00 (0–12)
NUTRIC score	2.92 ± 2.19	3.00 (0–8)
Duration of ventilation (days)	10.70 ± 10.56	6.50 (2–40)
ICU stay (days)	13.22 ± 12.88	8.00 (2–50)
Caloric requirement (indirect calorimetry, kcal/day)	1619.80 ± 253.82	1600.00 (1100–2300)
Caloric requirement (weight-based [25 kcal/kg/day], kcal/day)	1755.90 ± 242.48	1750.00 (1325–2300)
Caloric requirement (Faisy–Fagon equation, kcal/day)	1866.72 ± 215.81	1876.00 (974–2225)
Caloric requirement (PSU_m_ equation, kcal/day)	1631.09 ± 267.98	1589.24 (1199.24–2245.90)

**Table 2:** Laboratory data relating to the study participants

Variable	Mean ± SD
TLC	14.57 ± 6.94
Hemoglobin	10.42 ± 2.55
Platelet count	200.44 ± 111.14
PC	73.74 ± 17.15
International normalized ratio	1.27 ± 0.24
Urea	97.32 ± 68.68
Creatinine	2.47 ± 2.05
Na	138.0 ± 05.23
K	4.31 ± 0.82
ALT	53.20 ± 110.73
AST	52.84 ± 109.30
TB	1.39 ± 1.68
TLC	14.57 ± 6.94
Alb	2.73 ± 0.68
Ca	8.07 ± 1.27
Mg	2.09 ± 0.28
UA	7.31 ± 3.67
Phos	4.58 ± 1.80
LDL	79.84 ± 36.83
Chol	141.54 ± 47.84
TGs	105.99 ± 65.00

We found significant correlation between REE determined by indirect calorimetry and caloric requirements calculated by each of the weight-based, Faisy–Fagon, and PSU_m_ equations, with *P* values of ˂ 0.001, 0.001, and ˂ 0.001, respectively. However, there was also a significant difference between the measured REE and energy requirements estimated with the weight-based and Faisy–Fagon equations (*P* < 0.001 and 0.012, respectively). Energy expenditure estimation by PSU_m_ showed no significant difference from measured REE.

### Bias statistics

Bias was assessed by examining the 95% confidence interval (CI) of the difference between the estimated and measured metabolic rates [16]. This method captures the tendency of an equation to underestimate or overestimate the true value. If the 95% CI included zero, the equation was considered to be an unbiased estimator of the resting metabolic rate.

The precision of the predictive equations was assessed by examining the absolute differences between the estimated and measured resting metabolic rates as a percentage of the resting metabolic rates (also referred to as the root-mean-squared prediction error). Because this method uses the absolute difference, the canceling effect of underestimation and overestimation of the mean is eliminated. An estimate was considered precise if the root-mean- squared prediction error was ≤ 15% of the measurement.

Accuracy was assessed as the percentage of estimates that fell within 10% of the measured value, and by the incidence of large errors (> 15% above or below the measured rate).

Our results demonstrate that the Faisy–Fagon equation was biased towards overestimation of the caloric needs (95% CI 171–323 kcal/day) and was also imprecise, with a root-mean-squared prediction error > 15% (Table 3). By contrast, PSU_m_ was unbiased (95% CI − 48 to 71 kcal/day) and precise.

**Table 3:** Bias, accuracy, and precision of predictive equations relative to indirect calorimetry

Equation	Bias^a^ (kcal/day)	Accuracy^b^	Precision^c^	Incidence of large errors^d^
Weight-based (23 kcal/kg/day)	− 73.35–64.60	58.0%	8.75–15.46%	28%
PSU_m_	− 48.12–70.71	56.0%	7.53–13.12%	26.0%
Faisy–Fagon	170.50–323.34	30.0%	15.43–25.25%	54.0%

^a^Bias assessed by the 95% CI of the difference between estimated and measured metabolic rates. An interval including 0 indicates no bias

^b^Accuracy assessed as percentage of estimated values within 10% of corresponding measured values

^c^Precision assessed as 95% CI of root mean squared prediction errors considered precise if ≤ 15%

^d^Large errors were 15% above or below the measured value

Weight-based estimation at 25 kcal/kg/day was biased towards overestimation (95% CI 64.7–207.5 kcal/day), had fairly low precision (95% CI of prediction error 11.59–19.55%), 40% accuracy, and 38% incidence of large errors. However, weight-based estimation at 23 kcal/kg/day was unbiased, with greater precision and accuracy (Table 3), and with no significant difference between measured REE and calculated energy expenditure (data not shown).

**Fig. 1:**
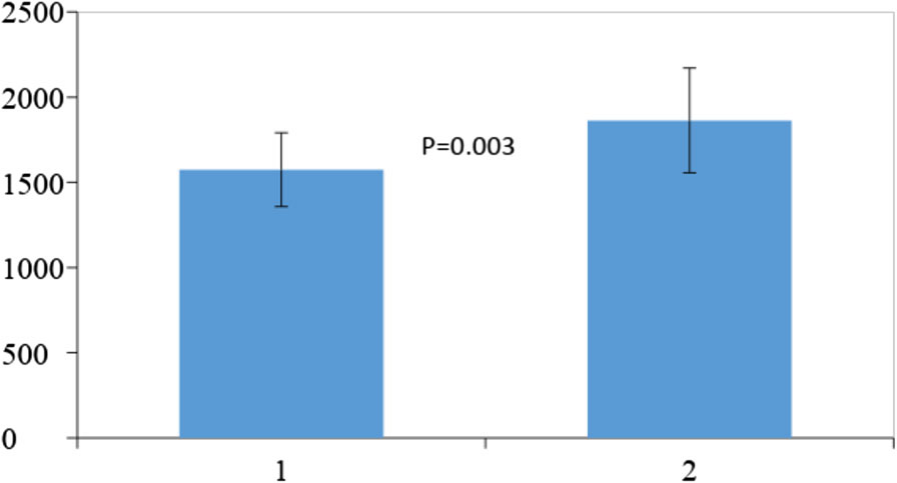
Bar chart for comparison between measured resting metabolic rate of patients who were weaned from mechanical ventilator and referred and those who failed to be freed from the machine and died

Next, APACHE II scores were significantly negatively correlated with measured REE (*P* = 0.031; Table 4). NUTRIC scores were not significantly correlated with measured REE, although both NUTRIC scores and REE were positively correlated with ICU length of stay (*P* = 0.011 and *P* = 0.015, respectively) and duration of ventilation (*P* = 0.001 and *P* = 0.012, respectively). Patients who were successively weaned from ventilation and referred from the ICU had significantly lower NUTRIC scores (*P* = 0.025) than those who were not. Mean REE was significantly higher (*P* = 0.003) in patients who survived and were referred from the ICU (1814.6 ± 273.4 kcal/day) than in those who died (1564.9 ± 222.1 kcal/day) as shown in (Fig. 1). REE was also significantly correlated with both height (*P* = 0.01) and weight (*P* < 0.001) (Table 4). Among the variables assessed by laboratory tests, platelet count, urea, creatinine, and phosphorus levels were significantly negatively correlated with REE (Table 4).

**Table 4:** Summary of parameters that correlated with indirect calorimetry

Parameter	Correlation coefficient	*P* value
Height	0.362	0.01
Weight	0.550	0.001
APACHE II score	− 0.306	0.031
Phosphorus	− 0.385	0.006
Urea	− 0.285	0.011
Creatinine	− 0.356	0.045
Platelet count	− 0.304	0.032
ICU length of stay	0.341	0.015
Duration of ventilation	0.917	0.012

## Discussion

In ventilated patients, calorimetry is often unavailable or inconvenient, making it important to derive equations that are predictive of REE. It is possible that results from these investigations can be extrapolated to non-intubated patients with similar degrees of illness in whom indirect calorimetry cannot be used.

The results indicated that in this population the Faisy–Fagon equation was biased towards overestimation, with a lower level of correlation with indirect calorimetry measurements than previously described [5]. Overestimation by this equation has also been described previously, including in groups of patients resembling our study population [7]. We also identified a lower level of precision with this equation than that determined in a previous study, with a 95% CI of the root-mean-squared prediction errors of 13–19% in our population and 15–25% previously.

In the population, estimations with the PSU_m_ equation showed significant correlation with measured REE, and this equation was unbiased and precise. In a previous study, the findings were similar, but with higher accuracy (67%) and lower incidence of large errors (13%) than in our population (56% and 26%, respectively) [17]. This difference may be attributed in part to non-homogeneity among their study population, only 42% of whom were medical patients.

In our population, weight-based estimation of energy requirement at a level of 25 kcal/kg/day produced poor results, whereas a similar approach using a level of 23 kcal/kg/day gave results that were comparable with those of PSU_m_ in terms of bias, precision, accuracy, and incidence of large errors. This finding suggests that a simple weight-based equation could be used instead of the more complex PSU_m_, in ventilated patients and in non-ventilated patients with a similar degree of illness.

Calculation of NUTRIC scores is a potential method for assessment of the nutritional needs of ICU patients, but in the population these scores did not show any correlation with measured REE. This finding is supported by previous results in which no difference was found between the outcomes of permissive underfeeding and standard enteral feeding in critically ill adults who were determined by NUTRIC scores to be either high or low nutritional risk [17]. These results suggest that NUTRIC scores cannot be used to differentiate between patients who may or may not benefit from different caloric dosing.

The study found that REE was significantly higher in patients who survived and left the ICU than in those who died. REE was also positively correlated with length of stay and duration of ventilation, suggesting that high REE was associated with survival of acute insults, prolonging ventilation and hospital length of stay. Similar results were obtained previously [18], with identification of a significant difference (*P* < 0.001) in REE between survivors (*n* = 846; mean REE = 1999.25 ± 485.37 kcal/day) and non-survivors (*n* = 325; mean REE = 1842.50 ± 497.48 kcal/day) at 60 days; however, no correlation was found between REE and 60-day mortality. These findings may be explained by the presence of ebb and flow phases. The ebb phase, which begins immediately after a traumatic shock, is characterized by a decrease in metabolic rate, decline in oxygen consumption and body temperature, and reduction in enzymatic activity. The flow phase follows and is marked by increased catabolism, with high oxygen consumption and elevation of energy expenditure [19].

Increased metabolism and catabolism in critically ill patients is considered an unavoidable shortcoming of severe illness, but it also represents an important survival mechanism, because the failure of the body to increase its metabolic rate leads to adverse outcomes. Decreased REE (a continuation of the ebb stage) may result from effects of sepsis and acute illness on mitochondri. In this model, patients who suffer less mitochondrial disturbance would have higher REEs and better outcomes than others with more severe injuries [20].

In the study population, the APACHE II score was negatively correlated with REE, suggesting that further studies could be conducted to determine whether there are any benefits to modulation of caloric intake according to patients’ APACHE scores. The study did not find a significant correlation between age and REE, in agreement with previous findings, [10] which may be partially explained by the effects of stress counteracting age-dependent reductions in metabolic rates. We found negative correlations between REE and several biochemical or cytological variables, including the level of phosphorus. This finding can be explained by mitochondrial injury. Phosphorus-containing compounds have important roles in cell structure, cellular metabolism, and regulation of subcellular processes; low phosphorus levels would therefore alter energy production [21]. In the recovery phase, increased physiological use of phosphorus leads to increased energy production and to reduction of systemic phosphorus levels, as occurs in refeeding syndrome.

Notably, there were some limitations to the study. The sample size was limited by the requirement for metabolic stability for measurement of REE. In clinical practice, most ventilated patients briefly satisfy this criterion, thereby enabling energy requirement to be estimated with a predictive equation, but not to be measured with indirect calorimetry. The study also had some strengths—to the best of our knowledge, it was the first prospective, single-center study to correlate REE in ventilated patients with the NUTRIC score and with patient outcomes. Moreover, the study population was homogeneous and included only mechanically ventilated medical patients.

## Conclusion

Weight-based caloric estimation is appealing because of its ease of use, applicability, and comparable or even superior accuracy compared with more sophisticated equations. Calculation of energy expenditure at values < 25 kcal/kg/day may be appropriate in patients with high APACHE II scores.

Notably, APACHE II scores were negatively correlated with REE, which could be helpful when calculating caloric needs.

REE was significantly higher in patients who were successfully weaned from ventilation and discharged alive than in those with worse outcomes. However, NUTRIC scores were not significantly correlated with REE and are unlikely to be useful for planning nutritional strategies for patients in the ICU.

## Data Availability

The datasets used and/or analyzed during the current study are available from the corresponding author on reasonable request.
